# Association of Vascular Risk Factors With β-Amyloid Peptide and Tau Burdens in Cognitively Unimpaired Individuals and Its Interaction With Vascular Medication Use

**DOI:** 10.1001/jamanetworkopen.2019.20780

**Published:** 2020-02-07

**Authors:** Theresa Köbe, Julie Gonneaud, Alexa Pichet Binette, Pierre-François Meyer, Melissa McSweeney, Pedro Rosa-Neto, John C. S. Breitner, Judes Poirier, Sylvia Villeneuve

**Affiliations:** 1 Department of Psychiatry, McGill University, Montreal, Quebec, Canada; 2 Studies on Prevention of Alzheimer’s Disease Centre, Douglas Mental Health University Institute, Montreal, Quebec, Canada; 3 Department of Neurology and Neurosurgery, McGill University, Montreal, Quebec, Canada; 4 McConnell Brain Imaging Centre, Montreal Neurological Institute, McGill University, Montreal, Quebec, Canada

## Abstract

**Importance:**

Vascular risk factors are associated with increased risk of Alzheimer disease (AD), but it is unclear whether there is a direct association of these risk factors with AD pathogenesis.

**Objectives:**

To assess the associations of vascular risk factors with AD pathogenesis in asymptomatic individuals, and to test whether this association is moderated among individuals who use vascular medications.

**Design, Setting, and Participants:**

This cross-sectional study used data from the Presymptomatic Evaluation of Experimental or Novel Treatments for Alzheimer Disease (PREVENT-AD) cohort of cognitively unimpaired individuals aged 55 to 82 years with a parental or multiple-sibling history of sporadic AD, who were recruited via advertisement from the greater Montreal, Quebec, Canada, metropolitan area. Participants were enrolled between September 9, 2011, to May, 3, 2017, and stratified by use vs no use of vascular medications. Data were analyzed July 1, 2018, to April 5, 2019.

**Main Outcomes and Measures:**

Principal analyses investigated associations of total, high-density lipoprotein, and low-density lipoprotein cholesterol levels, systolic and diastolic blood pressure, pulse pressure, and a combined vascular risk score (measured using the Framingham Coronary Risk Profile) with global β-amyloid peptide (Aβ) and entorhinal tau burden as measured by positron emission tomography (PET). Potential moderating associations of use of vascular medications with these associations were examined. Secondary similar analyses considered cerebrospinal fluid (CSF) Aβ1-42 and phosphorylated tau levels.

**Results:**

Among 215 participants (mean [SD] age, 62.3 [5.0] years; 161 [74.8%] women), 120 participants underwent PET, including 75 participants (62.5%) who were not using vascular medications, and 162 participants underwent CSF assessment, including 113 participants (69.8%) who were not using vascular medications. There was an overlap of 67 participants who underwent PET and CSF assessment. Interaction analyses showed that among participants not using vascular medications, higher Aβ deposition as measured by PET was associated with higher total cholesterol level (β = −0.002 [SE, 0.001]; *P* = .02), low-density lipoprotein cholesterol level (β = −0.002 [SE, 0.001]; *P* = .006), systolic blood pressure (β = −0.006 [SE, 0.002]; *P* = .02), pulse pressure (β = −0.007 [SE, 0.002]; *P* = .004), and Framingham Coronary Risk Profile score (β = −0.038 [SE, 0.011]; *P* = .001), but such associations were absent in participants who used vascular medications. Interactions were also found between vascular medication use and high-density lipoprotein cholesterol (β = −3.302 [SE, 1.540]; *P* = .03), low-density lipoprotein cholesterol (β = 1.546 [SE, 0.754]; *P* = .04), and Framingham Coronary Risk Profile score (β = 23.102 [SE, 10.993]; *P* = .04) on Aβ1-42 burden as measured in CSF. Higher Framingham Coronary Risk Profile scores were associated with reduced tau burden among participants using vascular medications but not among participants not using vascular medications (interaction, β = −0.010 [SE, 0.005]; *P* = .046).

**Conclusions and Relevance:**

These findings corroborate previously reported associations of vascular risk factors with Aβ burden but not tau burden. However, these associations were found only among individuals who were not using vascular medications. These results suggest that medication use or other control of vascular risk factors should be considered in Alzheimer disease prevention trials.

## Key Points

**Question:**

Does cardiovascular medication use moderate the association of vascular risk factors with Alzheimer disease pathogenesis as measured by β-amyloid peptide and tau burdens among individuals who are cognitively unimpaired?

**Findings:**

In this cross-sectional study of 215 middle- and late-aged adults who were cognitively unimpaired, use of vascular medications moderated an association of higher lipid levels, blood pressure, and combined vascular risk scores with increased brain β-amyloid peptide burden. Regarding tau burden, use of vascular medications moderated none but the association of combined vascular risk with higher entorhinal tau deposition.

**Meaning:**

This finding suggests that in individuals at risk for Alzheimer disease, treatment for common vascular risk factors may moderate or mask the associations of these factors with β-amyloid peptide burden.

## Teaser

This cross-sectional study examines the association of vascular risk factors with Alzheimer disease pathogenesis as measured by β-amyloid peptide and tau burdens in cognitively unimpaired individuals and further examines whether use of vascular medications moderates this association.

## Introduction

The neuropathological hallmarks of Alzheimer disease (AD) include cerebral β-amyloid peptide (Aβ) plaques and hyperphosphorylated tau neurofibrillary tangles. Vascular risk factors, such as dyslipidemia and hypertension, are thought to modify AD risk by promoting both cardiovascular disease and Aβ accumulation.^1^ While this dual vascular pathway hypothesis is attractive, results have been mixed. Cholesterol levels and blood pressure (BP) outside of reference ranges, as well as combined vascular risk scores, have been associated with increased Aβ burden in some studies,^2,3,4,5,6,7^ but other studies have had conflicting results.^8,9^ A few studies also found direct and indirect associations of vascular risk factors with increased tau burden,^8,10,11,12^ potentially moderated through Aβ burden; however, a 2009 study^13^ did not find these associations.

Such inconsistent results might be explained by potential moderation by vascular medications.^3,14^ Depending on treatment duration and type,^14,15^ the use of statins and antihypertensive drugs may be associated with providing protection against Aβ deposition.^16,17^ Participants using these vascular medications might even have experienced adverse effects of vascular risk factors over many years, but successful treatment preceding data collection could obscure the associations of these vascular risk factors with AD pathogenesis.

We examined whether use of vascular medications moderate the association of vascular risk factors (ie, cholesterol levels, BP, and a combined vascular risk score) with factors associated with AD pathogenesis (ie, Aβ and tau burdens) in middle- to late-aged individuals who were cognitively unimpaired and had a family history of AD. A first-degree family history of AD is associated with a 2- to 3-fold increased risk for AD,^18^ making individuals with such a family history of AD ideal for studying mechanisms associated with AD at an asymptomatic stage, which is the optimal time for prevention. Our principal hypothesis was that an association between vascular risk factors and AD pathogenesis would be stronger among individuals who did not use any medications to treat vascular risk factors (untreated cohort) than in individuals using vascular medications (treated cohort).

## Methods

### Participants and Study Design

Participants were recruited from the Presymptomatic Evaluation of Experimental or Novel Treatments for Alzheimer Disease (PREVENT-AD) cohort, an ongoing longitudinal observational study comprising a total sample size of 385 individuals.^19^ Inclusion criteria for PREVENT-AD were having parental or multiple-sibling history of AD-like dementia, being 60 years or older at enrollment or age 55 to 59 years if that was fewer than 15 years from the age of symptomatic dementia onset of a sibling or parent, having no major neurological diseases, and having unimpaired cognition. All participants exhibited unimpaired cognitive and functional scores on the Montreal Cognitive Assessment^20^ and the Clinical Dementia Rating^21^ before exhibiting unimpaired neuropsychological function on the Repeatable Battery for the Assessment of Neuropsychological Status.^22^ Fifteen individuals with ambiguous Repeatable Battery for the Assessment of Neuropsychological Status or Montreal Cognitive Assessment results were considered unimpaired after more extensive neuropsychological testing, as reviewed by neuropsychologists and physicians (including J.G., J.C.S.B, and S.V.). A flowchart of this study is presented in eFigure 1 in the Supplement.

These cross-sectional analyses considered a subsample of PREVENT-AD participants who had data on Aβ and tau burden, measured by positron emission tomography (PET) or cerebrospinal fluid (CSF) assessment. Analyses were conducted separately in participants who had PET or CSF assessment data. Participants were dichotomized further into those who did not report use of medications to remediate vascular risk factors (untreated cohort) vs those who reported using such medications, including lipid-lowering medications, antihypertensive medications, or both, at enrollment (treated cohort). A joint category of all participants who used vascular medications was created because dyslipidemia and hypertension often co-occur,^23,24^ and vascular medication may act on multiple pathways.^25^ All procedures followed the Strengthening the Reporting of Observational Studies in Epidemiology (STROBE) reporting guidelines for cross-sectional studies.

This study was approved by the McGill University Faculty of Medicine institutional review board. All participants received detailed study instructions and gave written informed consent prior to participation.

### Vascular Risk Factor Assessment

All participants underwent medical examinations, and nonfasting venous blood samples were taken at enrollment. Tests included assessment of plasma lipid concentrations and BP (eAppendix in the Supplement). Plasma levels of total, high-density lipoprotein (HDL), and low-density lipoprotein (LDL) cholesterol were measured using the CHOD-PAP method (Synchron LX, UniCel DxC 600/800 System and Synchron Systems Lipid Calibrator; Beckman Coulter). Blood pressure was assessed in a standardized procedure using an automatic sphygmomanometer (Connex ProBP 3400; Welch Allyn) while participants were seated. Pulse pressure was calculated as the difference between diastolic and systolic BP.

### Combined Vascular Risk Score

A variety of combined risk scores incorporating multiple vascular risk factors have been previously developed to estimate overall cardiovascular and coronary disease risk with the intent to detect individuals who are at increased risk with greater sensitivity than is possible by assessment of single risk factors.^26^ We used the Framingham Coronary Risk Profile (FCRP), a widely used index that estimates 10-year risk of coronary heart disease.^26^ The FCRP score is calculated as a sum of weighted measures of age, sex, systolic and diastolic BP, HDL and LDL cholesterol levels, smoking status, and diabetes status.^26^ Higher scores indicate greater risk.

### Assessments of Aβ and Tau Burdens

We performed PET scans using fluorine 18–labeled NAV4694 (NAV) for Aβ level and fluorine 18–labeled AV-1451 (Flortaucipir) for tau level estimation at the McConnell Brain Imaging Centre of the Montreal Neurological Institute, Montreal, Quebec, Canada, on a high-resolution PET scanner (Siemens). Static acquisition frames were obtained at 40 to 70 minutes after injection for Aβ and at 80 to 100 minutes after injection for tau. Structural magnetic resonance imaging (MRI) scans were T1-weighted and acquired on a 3-T Siemens Trio scanner at the Brain Imaging Centre of the Douglas Mental Health Institute, Montreal, using 2300 milliseconds for repetition time, 2.98 milliseconds for echo time, 176 slices, and slice thickness of 1 mm.

Cerebrospinal fluid samples were obtained by lumbar puncture in the morning under fasting conditions. Concentrations of Aβ1-42 and phosphorylated tau (pTau) were measured by enzyme-linked immunosorbent assay (INNOTEST; Fujirebio) as described previously.^27^ More information about PET and CSF assessment is available in the eAppendix in the Supplement.

### PET Processing

Data from PET were preprocessed using a standard pipeline.^28^ Briefly, 4-dimensional PET images were calculated for means and linearly coregistered to each individual’s T1-weighted images before being masked to exclude CSF binding and smoothed with a 6-mm^3^ Gaussian kernel. Individual T1-weighted images were segmented based on the Desikan-Killiany atlas using the semiautomated FreeSurfer processing stream version 5.3 (Martinos Center for Biomedical Imaging). Standardized uptake value ratios (SUVR) were computed for Aβ burden^29^ by dividing the tracer uptake by cerebellar gray matter uptake and for tau burden^30^ by dividing the tracer uptake by inferior cerebellar gray matter uptake. We restricted the region of interest analyses to FreeSurfer-derived AD-typical regions, in this case, weighted mean SUVRs from the frontal, temporal, parietal, and posterior cingulate cortices for Aβ quantification^29^ and from the entorhinal cortex for tau quantification.^31^

### Genotyping

Genomic DNA was extracted from whole blood, and apolipoprotein E (*APOE*) genotype was determined using the PyroMark Q96 pyrosequencer (Qiagen), as described previously.^27^ Participants were classified as *APOE *ε4 carriers (ie, those who had 1-2 ε4 alleles) or noncarriers.

### Statistical Analysis

Group differences between the treated vs untreated cohorts in the PET and in the CSF assessment groups were tested using unpaired *t* tests for normally distributed continuous variables, Mann-Whitney *U* tests for nonnormally distributed continuous variables, or χ^2^ tests for categorial variables. Individuals with missing values were excluded from respective analyses.

To test for the main association of vascular risk factors with AD brain pathogenesis as measured by Aβ or tau deposition, we performed multiple linear regression analyses in untreated and treated cohorts combined. Models included age, sex, vascular medication status (ie, using medication or not), and time difference between vascular risk factor assessments and undergoing PET as covariates. A second model tested for statistical interaction between vascular risk factors and vascular medication status in association with AD brain pathogenesis, retaining the same covariates as in the first model. Finally, we examined associations of vascular risk factors with AD brain pathogenesis separately for treated and untreated cohorts using independent linear regressions and the same covariates as in the second model. On observing a significant interaction, subgroup analyses were interpreted.

To investigate whether imaging findings could be reproduced using CSF biomarkers, we performed the same multiple regression and within-group analyses using CSF Aβ1-42 and pTau levels as dependent variables. While PET and CSF Aβ and tau biomarkers are known to be correlated, they do not capture the exact same pathological components (soluble vs nonsoluble); thus, PET and CSF may be considered as complementary markers.^32,33^

The mean (range) time delays between assessments of vascular risk factors at enrollment and assessment of Aβ and tau were 45 (3-75) months for participants who underwent PET and 11 (0-62) months for participants who underwent CSF assessment. Vascular risk factors were always assessed prior to PET or CSF assessment. Although we did not expect noteworthy changes in SUVRs as measured by PET within 45 months,^34^ we adjusted all statistical models for the time between vascular risk factors and PET or CSF assessment. To explore potential modifying associations of *APOE *ε4 status, we performed identical analyses that included *APOE *ε4 status as an additional covariate in the models.

Under the assumption that both lipid and BP biomarkers would be associated with AD pathogenesis, we ran an exploratory regression model including both types of risk factors to test whether they were independently associated with AD pathogenesis. Supplementary analyses included linear regression to test whether longer treatment durations for dyslipidemia or hypertension were associated with reduced AD pathological burden. Treatment duration before enrollment in PREVENT-AD was reported by participants through an online questionnaire (response rate, 88%).

Analyses were performed with SPSS statistical software version 24.0 (IBM Corp). Two-tailed *P* values less than .05 were considered statistically significant. Analyses were not corrected for multiple comparisons because of complementarity of the vascular risk factor measures. Individual results should therefore be interpreted with caution.

## Results

### Participant Characteristics

The study sample included 215 participants (mean [SD] age, 62.3 [5.0], years; 161 [74.9%] women), 87 of whom (40.5%) were *APOE *ε4 carriers. Among 215 participants, 120 participants underwent PET, including 75 participants (62.5%) who were not using vascular medications, and 162 participants underwent CSF assessment, including 113 participants (69.8%) who were not using vascular medications. There was an overlap of 67 participants who underwent PET and CSF assessment. Among both groups, a total of 69 participants (mean [SD] age, 63.4 [4.5] years; 47 [68.1%] women) used vascular medications, and 146 participants (mean [SD] age, 61.8 [5.1] years; 114 [78.1%] women) did not use vascular medications. Compared with participants in the untreated cohort, participants in the treated cohort had lower mean (SD) concentrations of total cholesterol (217.43 [33.2] mg/dL vs 189.31 [39.1] mg/dL; *P* < .001) and LDL cholesterol (124.96 [27.29] mg/dL vs 95.75 [36.20] mg/dL; *P* < .001) but higher systolic BP (123.77 [14.4] mm Hg vs 131.67 [12.7] mm Hg; *P* < .001) and pulse pressure (50.88 [10.8] mm Hg vs 55.81 [12.7] mm Hg; *P* = .003) (to convert cholesterol to millimoles per liter, multiply by 0.0259). Levels of Aβ and tau were comparable in participants who underwent PET and those who underwent CSF assessment regardless of treatment status. Complete participant characteristics are presented in Table 1.

**Table 1.** Participant Characteristics

Characteristic	Participants, Mean (SD) [Range] (N = 215)^a^					
	Underwent PET (n = 120)			Underwent CSF Assessment (n = 162)		
	Untreated Cohort (n = 75)	Treated Cohort (n = 45)	*P* Value	Untreated Cohort (n = 113)	Treated Cohort (n = 49)	*P *Value
Women, No. (%)	56 (74.7)	33 (73.3)	.52^b^	88 (77.9)	29 (59.2)	.02^b^
Age, y	63 (4.8) [55-78]	64 (4.4) [57-73]	.045^c^	62 (5.2) [55-82]	63 (4.6) [55-74]	.05^d^
Education, y	15 (3.2) [7-24]	15 (3.4) [7-24]	.51^d^	16 (3.0) [10-27]	14 (3.1) [7-20]	.09^c^
MOCA score	28.2 (1.5) [24-30]	28.2 (1.6) [24-30]	.86^c^	27.9 (1.5) [23-30]	28.2 (1.5) [25-30]	.39^c^
*APOE *ε4 carrier, No. (%)	28 (37.3)	21 (46.7)	.31^b^	40 (35.4)	24 (49.0)	.12^b^
Plasma cholesterol level, mg/dL						
Total	216.13 (36.0) [135.35-320.96]^e^	188.37 (37.4) [127.61-278.42]	<.001^c^	217.76 (32.5) [143.08-320.96]^e^	186.42 (39.1) [112.14-274.56]^e^	<.001^c^
HDL	60.31 (16.1) [28.62-99.00]^e^	55.56 (13.2) [34.42-96.29]	.10^c^	63.15 (17.1) [28.62-121.81]^e^	57.55 (15.4) [34.42-110.60]^e^	.05^c^
LDL	126.25 (28.1) [66.51-223.13]^f^	94.84 (38.2) [38.28-194.12]^g^	<.001^c^	125.21 (28.0) [61.49-223.13]^h^	91.65 (33.4) [38.28-167.44]^h^	<.001^c^
Arterial blood pressure, mm Hg						
Systolic	125.5 (13.3) [100-158]	134.2 (12.4) [107-168]	.001^c^	123.6 (14.9) [92-162]	131.0 (12.3) [110-164]	.003^c^
Diastolic	73.2 (7.5) [60-94]	75.5 (8.3) [60-93]	.12^d^	73.0 (8.7) [52-98]	75.4 (8.8) [58-93]	.09^d^
Pulse pressure, mm Hg	52.3 (10.8) [24-80]	58.7 (12.7) [35-88]	.004^c^	50.8 (10.9) [26-80]	55.7 (12.2) [32-84]	.01^c^
FCRP score	5.9 (2.6) [1-12]^g^	6.7 (2.9) [1-12]	.16^d^	5.7 (2.5) [0-12]^h^	5.7 (2.3) [1-11]^h^	.97^d^
Aβ, [^18^F]NAV4694 SUVR, median (IQR) [range]	1.2 (1.14-1.31) [1.1-2.3]	1.22 (1.15-1.33) [1.0-2.8]	.68^d^	NA	NA	NA
Tau, [^18^F]AV-1451 SUVR, median (IQR) [range]	1.05 (0.99-1.13) [0.7-1.7]	1.04 (0.99-1.16) [0.9-1.4]^e^	.92^d^	NA	NA	NA
Aβ1-42, pg/mL	NA	NA	NA	1192 (275) [479-1760]^i^	1138 (261) [501-1568]^j^	.27^c^
Phosphorylated tau, pg/mL	NA	NA	NA	47.5 (16.0) [12.1-98.2]^e^	48.8 (17.9) [17.8-114.4]	.85^d^
Medication use, No. (%)						
Lipid-lowering drugs	NA	17 (38)	NA	NA	21 (43)	NA
Antihypertensive drugs	NA	14 (31)	NA	NA	12 (24)	NA
Lipid-lowering and antihypertensive drugs	NA	14 (31)	NA	NA	16 (33)	NA
Duration of medication use, y	NA	10.4 (8.7) [1-36]^g^	NA	NA	9.0 (5.7) [1-21]^k^	NA

Abbreviations: Aβ, β-amyloid; CSF, cerebrospinal fluid; ^18^F, fluorine 18; FCRP, Framingham Coronary Risk Profile; HDL, high-density lipoprotein; IQR, interquartile range; LDL, low-density lipoprotein; MOCA, Montreal Cognitive Assessment; NA, not applicable; PET, positron emission tomography; SUVR, standard uptake value ratio.

SI conversion factor: To convert cholesterol to millimoles per liter, multiply by 0.0259.

^a^ There were 67 participants (31%) who underwent both PET and CSF assessments and were included in both analyses, including 42 participants in the untreated cohorts and 25 participants in the treated cohorts.

^b^ Calculated with χ^2^ test.

^c^ Calculated with unpaired *t* test.

^d^ Calculated with Mann-Whitney *U* test.

^e^ Missing data for 1 participant.

^f^ Missing data for 3 participants.

^g^ Missing data for 2 participants.

^h^ Missing data for 4 participants.

^i^ Missing data for 15 participants.

^j^ Missing data for 5 participants.

^k^ Missing data for 8 participants.

Participants were generally in good health, but nevertheless presented a wide range of vascular risk factors. The predominant vascular risk factors recorded in the total cohort were total cholesterol level more than 200 mg/dL (124 participants [57.9%]), LDL cholesterol level more than 130 mg/dL (72 participants [34.0%]), systolic BP 130 mm Hg or higher (83 participants [38.6%]), diastolic BP 80 mm Hg or higher (66 participants [30.1%]) and body mass index (calculated as weight in kilograms divided by height in meters squared) more than 30 (47 participants [22.0%]). Approximately 4% of participants had diabetes, and approximately 3% of participants reported current smoking.

### Associations of Vascular Risk Factors With Global Aβ and Entorhinal Tau SUVR Measured by PET

**Figure 1.**
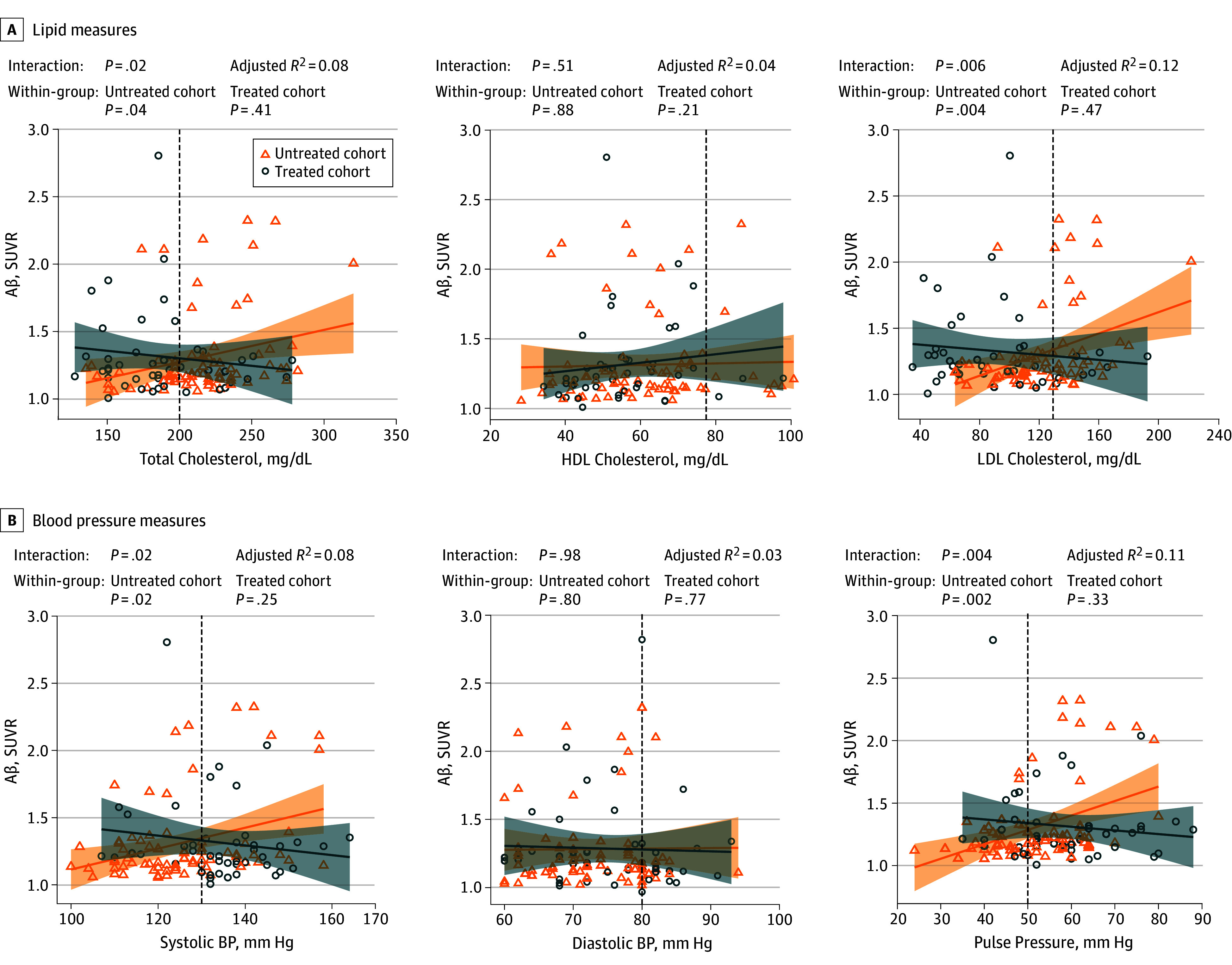
Associations of Vascular Risk Factors and β-Amyloid Peptide (Aβ) Burden as Measured by Positron Emission Tomography Concentrations of vascular risk factors are plotted against global cerebral Aβ standard uptake value ratios (SUVRs) measured with position emission tomography. Multiple linear regression analyses (solid lines) were controlled for age, sex, and time differences. Shaded area indicates 95% CI; vertical dotted line, threshold for reference value of the vascular risk factor; BP, blood pressure; HDL, high-density lipoprotein; and LDL, low-density lipoprotein. To convert cholesterol to millimoles per liter, multiply by 0.0259.

Among participants who underwent PET, no association of vascular risk factors, either as individual factors or combined as FCRP score, was found with global cerebral Aβ deposition. However, interaction analyses showed that among participants not using vascular medications, higher Aβ deposition as measured by PET was associated with higher total cholesterol (unstandardized β = −0.002 [SE, 0.001]; *P* = .02), low-density lipoprotein cholesterol (β = −0.002 [SE, 0.001]; *P* = .006), systolic BP (β = −0.006 [SE, 0.002]; *P* = .02), pulse pressure (β = −0.007 [SE, 0.002]; *P* = .004), and Framingham Coronary Risk Profile score (β = −0.038 [SE, 0.011]; *P* = .001), but such associations were absent in participants who used vascular medications (Figure 1 and Table 2). Specifically, in untreated participants, a 50-mg/dL increase in total cholesterol level was associated with an increase of 0.10 in Aβ SUVR, and a 50-mg/dL increase in LDL cholesterol level was associated with an increase of 0.20 in Aβ SUVR. A 10–mm Hg increase in systolic BP was associated with a 0.06 increase in Aβ SUVR, and a 10–mm Hg increase in pulse pressure was associated with a 0.10 increase in Aβ SUVR. A 1-unit increase in FCRP score was associated with a 0.03 increase in Aβ SUVR. No similar associations were found in the treated cohort of participants who underwent PET. These results remained largely unchanged when *APOE *ε4 status was added as an additional covariate (eTable 1 in the Supplement).

**Table 2.** Associations of Vascular Risk Factors With Global Cerebral Aβ Burden Measured by Positron Emission Tomography

Variable	Aβ Burden, Unstandardized β (SE) [*P* Value]						
	Cholesterol Level			Blood Pressure		Pulse Pressure	FCRP Score
	Total	HDL	LDL	Systolic	Diastolic		
Model 1							
Age	0.014 (0.006) [.02]	0.015 (0.006) [.02]	0.015 (0.006) [.02]	0.013 (0.006) [.047]	0.015 (0.006) [.02]	0.012 (0.007) [.06]	0.015 (0.007) [.03]
Sex	−0.058 (0.067) [.39]	−0.061 (0.069) [.38]	−0.090 (0.069) [.20]	−0.036 (0.065) [.58]	−0.041 (0.065) [.54]	−0.034 (0.065) [.60]	−0.080 (0.072) [.27]
Time difference	−0.002 (0.002) [.15]	−0.002 (0.002) [.14]	−0.002 (0.002) [.18]	−0.002 (0.002) [.12]	−0.002 (0.002) [.12]	−0.002 (0.002) [.12]	−0.003 (0.002) [.11]
Medication	0.009 (0.064) [.89]	−0.008 (0.061) [.89]	0.033 (0.068) [.63]	−0.033 (0.062) [.60]	−0.015 (0.060) [.80]	−0.032 (0.061) [.60]	−0.008 (0.062) [.90]
Vascular risk factor	0.001 (0.001) [.28]	0.002 (0.002) [.38]	0.001 (0.001) [.17]	0.002 (0.002) [.34]	−0.001 (0.004) [.87]	0.003 (0.003) [.23]	0.003 (0.012) [.82]
Model 2: interaction of vascular risk factor × medication^a^	−0.002 (0.001) [.02]	0.001 (0.002) [.51]	−0.002 (0.001) [.006]	−0.006 (0.002) [.02]	<−0.001 (0.004) [.98]	−0.007 (0.002) [.004]	−0.038 (0.011) [.001]
Untreated cohort							
Age	0.019 (0.007) [.01]	0.020 (0.008) [.009]	0.019 (.007) [.01]	0.016 (0.007) [.04]	0.020 (0.007) [.008]	0.014 (0.007) [.047]	0.014 (0.008) [.09]
Sex	0.018 (0.082) [.82]	0.063 (0.089) [.48]	0.021 (0.081) [.80]	0.078 (0.078) [.32]	0.060 (0.081) [.46]	0.100 (0.077) [.20]	0.021 (0.084) [.80]
Time difference	−0.002 (0.002) [.18]	−0.002 (0.002) [.19]	−0.002 (0.002) [.30]	−0.002 (0.002) [.25]	−0.002 (0.002) [.18]	−0.002 (0.002) [.30]	−0.003 (0.002) [.13]
Vascular risk factor	0.002 (0.001) [.04]	<−0.001 (0.002) [.88]	0.004 (0.001) [.004]	0.006 (0.003) [.02]	−0.001 (0.005) [.80]	0.010 (0.003) [.002]	0.033 (0.015) [.03]
Treated cohort							
Age	0.006 (0.011) [.56]	0.007 (0.011) [.50]	0.009 (0.011) [.43]	0.012 (0.012) [.32]	0.006 (0.011) [.58]	0.012 (0.012) [.33]	0.014 (0.011) [.20]
Sex	−0.175 (0.112) [.13]	−0.220 (0.111) [.05]	−0.275 (0.117) [.02]	−0.192 (0.109) [.09]	−0.194 (0.111) [.09]	−0.188 (0.109) [.09]	−0.205 (0.122) [.10]
Time difference	−0.004 (0.003) [.24]	−0.003 (0.003) [.30]	−0.005 (0.003) [.12]	−0.003 (0.003) [.37]	−0.003 (0.003) [.31]	−0.002 (0.003) [.40]	−0.004 (0.003) [.14]
Vascular risk factor	−0.001 (0.001) [.41]	0.005 (0.004) [.21]	−0.001 (0.001) [.47]	−0.005 (0.004) [.25]	−.002 (0.006) [.77]	−0.004 (0.004) [.33]	−0.033 (0.019) [.09]

Abbreviations: Aβ, β-amyloid peptide; FCRP, Framingham Coronary Risk Profile; HDL, high-density lipoprotein; LDL, low-density lipoprotein.

^a^ Adjusted for all covariates included in model 1.

When included in the same regression model, we found independent associations of Aβ burden with LDL cholesterol level (β = 0.003 [SE, 0.001], *P* = .006) and pulse pressure (β = 0.010 [SE, 0.003], *P* = .002) in the untreated cohort. Within the treated group, there were no associations of treatment duration with Aβ SUVR after adjusting for age and sex (β = −0.007 [SE, 0.004]; *P* = .09).

In contrast to Aβ, there were no main associations of single vascular risk factors with tau deposition, and there were no interactions between single vascular risk factors and vascular medication associated with tau deposition in the entorhinal cortex. An interaction was found between FCRP score and medication use associated with entorhinal tau*.* Within-group analyses of the treated cohort suggested that a 1-unit increase in the FCRP score was associated with a 0.02 SUVR decrease in entorhinal tau (β = −0.010 [SE, 0.005]; *P* = .046) (Table 3; eFigure 2 in the Supplement). This isolated association lost significance with adjustment for *APOE *ε4 status (eTable 2 in the Supplement).

**Table 3.** Associations of Vascular Risk Factors With Entorhinal Tau Burden as Measured by Positron Emission Tomography

Variable	Tau Burden, Unstandardized β (SE) [*P* Value]						
	Cholesterol Level			Blood Pressure		Pulse Pressure	FCRP Score
	Total	HDL	LDL	Systolic	Diastolic		
Model 1							
Age	0.007 (0.003) [.008]	0.008 (0.003) [.006]	0.008 (0.003) [.006]	0.008 (0.003) [.01]	0.008 (0.003) [.006]	0.007 (0.003) [.01]	0.010 (0.003) [.001]
Sex	0.018 (0.029) [.54]	<0.001 (0.030) [>.99]	0.009 (0.031) [.76]	0.016 (0.029) [.57]	0.017 (0.029) [.56]	0.017 (0.029) [.56]	0.023 (0.031) [.47]
Time difference	<−0.001 (0.001) [.93]	<0.001 (0.001) [.97]	<0.001 (0.001) [.86]	<−0.001 (0.001) [.96]	<−0.001 (0.001) [.96]	<−0.001 (0.001) [.97]	<−0.001 (0.001) [.96]
Medication	−0.012 (0.028) [.68]	−0.006 (0.026) [.83]	−0.011 (0.030) [.72]	−0.010 (0.027) [.72]	−0.009 (0.027) [.74]	−0.012 (0.027) [.66]	−0.005 (0.027) [.85]
Vascular risk factor	<−0.001 (<0.001) [.95]	0.002 (0.001) [.07]	<−0.001 (<0.001) [.93]	<0.001 (0.001) [.94]	−0.001 (0.002) [.66]	<0.001 (0.001) [.82]	−0.009 (0.005) [.09]
Model 2: interaction of vascular risk factor × medication^a^	<−0.001 (<0.001) [.56]	0.001 (0.001) [.30]	−0.001 (0.001) [.35]	−0.001 (0.001) [.35]	<0.001 (0.002) [.93]	−0.001 (0.001) [.35]	−0.010 (0.005) [.046]
Untreated cohort							
Age	0.007 (0.004) [.06]	0.007 (0.004) [.06]	0.007 (0.004) [.05]	0.006 (0.004) [.08]	0.007 (.004) [.048]	0.006 (0.004) [.10]	0.007 (0.004) [.07]
Sex	0.061 (0.040) [.13]	0.056 (0.042) [.19]	0.059 (0.041) [.15]	0.063 (0.039) [.11]	0.061 (0.038) [.12]	0.067 (0.039) [.09]	0.061 (0.041) [.14]
Time difference	<0.001 (0.001) [.89]	<0.001 (0.001) [.86]	<0.001 (0.001) [.84]	<0.001 (0.001) [.80]	<0.001 (0.001) [.85]	<0.001 (0.001) [.75]	<0.001 (0.001) [.86]
Vascular risk factor	<0.001 (<0.001) [.96]	<0.001 (0.001) [.72]	<0.001 (0.001) [.62]	0.001 (0.001) [.51]	−0.001 (0.002) [.76]	0.002 (0.002) [.30]	<0.001 (0.007) [.97]
Treated cohort							
Age	0.009 (0.004) [.03]	0.010 (0.004) [.02]	0.011 (0.004) [.01]	0.012 (0.005) [.01]	0.009 (0.004) [.04]	0.012 (0.005) [.02]	0.014 (0.004) [.001]
Sex	−0.056 (0.042) [.19]	−0.081 (0.038) [.04]	−0.089 (0.043) [.046]	−0.057 (0.040) [.16]	−0.060 (0.041) [.15]	−0.057 (0.041) [.17]	−0.044 (0.042) [.30]
Time difference	−0.001 (0.001) [.52]	−0.001 (0.001) [.49]	−0.002 (0.001) [.17]	−0.001 (0.001) [.62]	−0.001 (0.001) [.53]	<0.001 (0.001) [.64]	−0.001 (0.001) [.17]
Vascular risk factor	<0.001 (0.001) [.76]	0.003 (0.001) [.01]	<0.001 (<0.001) [.46]	−0.002 (0.002) [.24]	−0.001 (0.002) [.64]	−0.001 (0.002) [.42]	−0.019 (0.007) [.006]

Abbreviations: FCRP, Framingham Coronary Risk Profile; HDL, high-density lipoprotein; LDL, low-density lipoprotein.

^a^ Adjusted for all covariates included in model 1.

### Associations of Vascular Risk Factors With Aβ1-42 and pTau Levels Measured by CSF Assessment 

**Figure 2.**
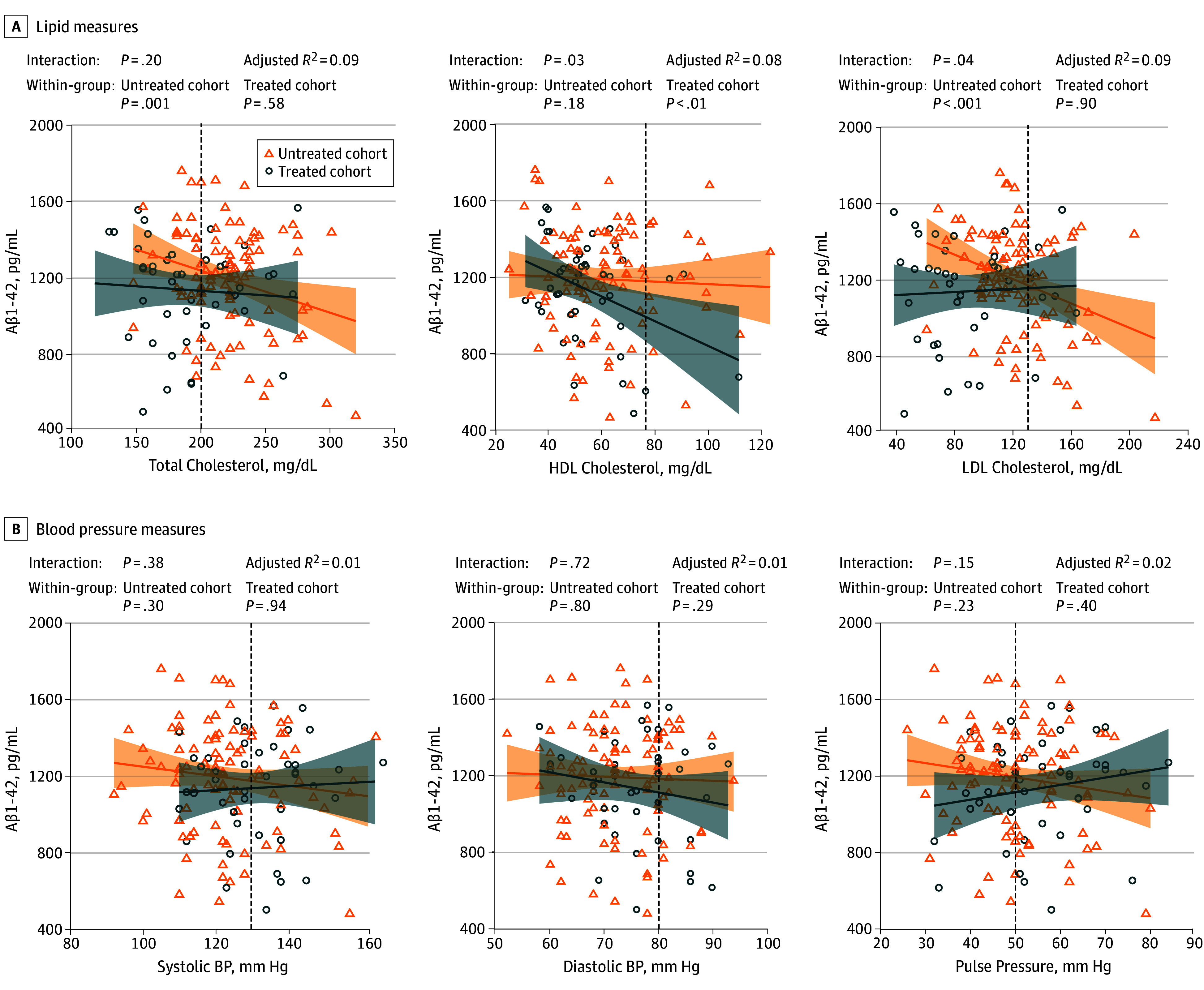
Associations of Vascular Risk Factors With β-Amyloid Peptide (Aβ) Burden as Measured by Cerebrospinal Fluid Assessment Concentrations of vascular risk factors are plotted against Aβ1-42 levels measured in cerebrospinal fluid. Multiple linear regression analyses (solid lines) were controlled for age, sex, and time differences between vascular risk factor and cerebrospinal fluid assessments. Shaded area indicates 95% CI; vertical dotted line, threshold for reference value of the vascular risk factor; BP, blood pressure; HDL, high-density lipoprotein; and LDL, low-density lipoprotein. To convert cholesterol to millimoles per liter, multiply by 0.0259.

Comparable analyses using CSF assessments found negative associations of lower Aβ1-42 concentration, representing higher Aβ burden, with higher total cholesterol (β = −2.010 [SE, 0.641], *P* = .002) and HDL cholesterol (β = −3.525 [SE, 1.474]; *P* = .02) levels. Interactions were also found between vascular medication use and high-density lipoprotein cholesterol (β = −3.302 [SE, 1.540]; *P* = .03), low-density lipoprotein cholesterol (β = 1.546 [SE, 0.754]; *P* = .04), and Framingham Coronary Risk Profile score (β = 23.102 [SE, 10.993]; *P* = .04) on Aβ1-42 burden as measured in CSF. More specifically, a 50-mg/dL increase in LDL cholesterol was associated with a 164-pg/mL decrease in Aβ1-42 levels (thus higher Aβ burden) in the untreated cohort. No significant associations were found between FCRP score and Aβ1-42 in the treated or untreated cohorts. In the treated cohort, we found that a 50-mg/dL increase in HDL cholesterol level was associated with a 393-pg/mL decrease in Aβ1-42, reflecting higher Aβ burden (Figure 2; eTable 3 in the Supplement). After adjustment for *APOE *ε4 status, only the associations with LDL cholesterol level remained significant within the untreated cohort (eTable 4 in the Supplement).

No association was found between treatment duration and Aβ1-42 concentration after adjusting for age and sex (β = 0.339 [SE, 7.971]; *P* = .97). Consistent with the PET data, no main associations of vascular risk factors and no interactions between vascular risk factors and vascular medication use were found with pTau levels as measured by CSF assessment (eFigure 3 and eTable 5 in the Supplement) or after correction for *APOE *ε4 status (eTable 6 in the Supplement).

## Discussion

This cross-sectional study found that cardiovascular medication use moderated the associations of vascular risk factors with Aβ burden among middle- to late-aged individuals who were cognitively unimpaired but at risk of AD. Specifically, we observed associations of higher total and LDL cholesterol levels, systolic BP, pulse pressure, and a composite of vascular risk factors, FCRP score, with increased cerebral Aβ deposition in individuals who did not use vascular medications. Importantly, these associations were not present in individuals who used vascular medications. The associations were not significant when participants were pooled together regardless of their medication status. The findings with LDL cholesterol levels and FCRP score were replicated when Aβ burden was measured using CSF Aβ1-42 levels. We did not observe associations between individual vascular risk factors and cerebral or CSF tau accumulation, but vascular medication use showed an interaction with the association of FCRP score with tau deposition as measured by PET, such that higher FCRP scores were associated with reduced tau deposition in the treated cohort.

While some neuroimaging and autopsy studies have revealed no association of vascular health with Aβ deposition,^8,35^ others have shown detrimental associations of higher cholesterol levels^2^ and BP^4,36^ with Aβ accumulation. Our findings may explain these inconsistencies by showing that vascular medication use moderated those associations such that, in our sample, such associations were detectable only in untreated individuals. Similarly, a 2014 study^6^ reported that vascular risk factor burden and Aβ were associated with reduced cortical thickness, particularly in individuals who did not use cholesterol-lowering medications, compared with individuals who were treated for hyperlipidemia.

Our finding that lipid-lowering and antihypertensive medications moderated the associations of vascular risk factors with Aβ burden could have clinical implications. It has been suggested that cardiovascular medication might reduce AD risk by lowering arterial stiffness, leading to increased cerebral blood flow and Aβ clearance.^16,37^ However, results from a randomized clinical trial^38^ and a meta-analysis of cohort studies^39^ have been mixed. Other factors, such as treatment duration, participants’ age at treatment onset, and medication type, appear to be important to the association of vascular risk factor burden with AD-related end points.^6,15^ Interestingly, the mean Aβ burden was similar in our treated and untreated cohorts. An alternative explanation for why an association of vascular risk factors with Aβ burden was not observed in treated participants might be that vascular risk factors were successfully lowered in most treated participants, but Aβ deposition had started before treatment onset. Figure 1 suggests this, at least with lipid levels: the treated cohort had cholesterol levels within reference ranges, but they may still have experienced adverse effects of their hypercholesterolemia before it was treated. This question may depend also on their medication type, timing, and duration.

The elevated AD risk in our study participants should render them well suited for analyses examining the associations of vascular risk factors with AD pathogenesis. Different time points of assessment of vascular risk factors in life might also influence their association with Aβ burden. For example, vascular risk factors in midlife (age ≤70 years) may be associated with increased AD risk and pathogenesis.^5,40^ Our results from a cohort of participants in middle to late age (mean age, 62 years) support these findings.

Although dyslipidemia and hypertension often co-occur,^23,24^ including both LDL cholesterol level and pulse pressure as factors associated with cerebral Aβ burden in a single regression model did not change our findings, suggesting that these factors were independently associated with the outcome. However, the cross-sectional nature of our analyses makes it possible that the observed associations were inversely related, so that Aβ burden might be associated with altered intracellular vesicle trafficking and metabolic cholesterol homeostasis^41^ or vasopressor actions.^42^

The strongest known genetic AD risk factor, *APOE *ε4, has been found to be associated with lipid metabolism,^43^ vasculature,^44^ Aβ deposition,^45^ and moderating the associations of vascular risk factors with AD pathogenesis.^3,46^ However, some studies have reported conflicting results.^7,8,40^ In our study, associations of vascular risk factors with Aβ burden measured using PET remained after correction for *APOE *ε4 status, as has been reported previously.^7,40^ However, findings regarding CSF Aβ1-42 levels were only partially independent of *APOE *ε4 status, suggesting that a potential interaction between genetics and vascular risk factors associated with AD pathogenesis should be further investigated.

It has been argued that composite vascular risk scores are more sensitive measures for detecting associations of vascular risk factors with Aβ burden,^6,7,40^ although this has not been consistently confirmed.^12,47^ In our study, the combined FCRP score, LDL cholesterol level, and pulse pressure were the strongest factors associated with presymptomatic AD pathogenesis, but all of these factors had similar associations with Aβ burden, which does not suggest superior sensitivity of the FCRP score compared with single vascular risk factors. One reason for these results might be that our study sample included very few individuals with diabetic comorbidities or smokers, which are both included in the calculation of FCRP score.

With regard to tau, two 2019 studies of older adults who were cognitively unimpaired but who had a mean age 10 years older than participants from the PREVENT-AD cohort reported that higher vascular risk factor burden was associated with increased tau burden in the brain^12^ and CSF.^48^ We found only 1 interaction between vascular medication use and the FCRP score associated with cerebral tau burden, which unexpectedly suggested that higher vascular risk factor burden was associated with lower tau deposition in the treated cohort. This isolated result could suggest that tau burden is less sensitive to vascular risk factors than Aβ burden and that a combined vascular risk score may be needed to show any association. However, we should note that the levels of cerebral tau in our relatively young cohort were fairly low compared with cerebral Aβ levels; therefore, associations of vascular risk factors and tau burden may have been more difficult to detect. Furthermore, the association of vascular risk factors with tau was no longer significant when we controlled for *APOE *ε4 status, drawing into question the strength of this individual association. Further investigations are needed to better understand whether and how vascular risk factors are associated with tau burden in the early AD continuum, and these inquiries should consider confounding factors, such as medication use.

### Strengths and Limitations

Our study has some strengths, including its reliance on middle- to late-aged individuals at risk for AD, thereby perhaps exposing events in the initial accumulation of Aβ burden, that is, a time when vascular risk factors may remain a promising target for disease prevention. The fact that PET findings were substantiated, at least in part, by observations in CSF biomarkers associated with AD lends further credence to the association of vascular risk factors with Aβ burden in such individuals. Although we adjusted all models for the time difference between assessments of vascular risk factors and assessments of biomarkers associated with AD pathogenesis, the discord in findings with PET and CSF assessment might reflect additional progression of AD stage at the time of PET assessment,^49^ which was conducted a mean of 34 months after the CSF assessment. Different PET and CSF findings might also reflect the measurement of different components of Aβ burden.^49^ The prevalence of vascular risk factors in our cohort was generally comparable to that of the general US population,^50^ except that current smoking status was approximately 10-fold lower in the PREVENT-AD cohort, suggesting that our results are extendible to further populations.

Our study also has some limitations. We cannot exclude the possibility that participants in the treated cohort may have had healthier lifestyles (eg, more regular exercise, maintaining healthy weight, reduced alcohol consumption and smoking) because of an increased awareness of health concerns. Such health consciousness may also have influenced our results. Other limitations of the study include its cross-sectional design and that BP values were assessed from only 1 measurement instead of determining a mean of multiple measurements at enrollment. Studies with larger sample sizes are warranted to investigate sex-specific associations of vascular risk factors with AD pathogenesis.

## Conclusions

The findings of this cross-sectional study suggest that an individual’s use of vascular medications is an important consideration when studying any association of vascular risk factors and AD pathogenesis. Our findings also suggest the importance of targeting both systemic vascular burden and Aβ burden in interventional studies of healthy individuals at risk of AD. Given the current lack of effective AD treatments, the identification of modifiable risk factors associated with development of presymptomatic AD trajectories should be of considerable interest for AD prevention research.

## Notes

Supplement. **eAppendix.** Supplementary Methods **eFigure 1. **Study Flow Chart **eFigure 2. **Associations of Vascular Risk Factors With Tau Burden as Measured by Positron Emission Tomography in Treated and Untreated Cohorts **eFigure 3. **Associations of Vascular Risk Factors With Phosphorylated Tau as Measured by Cerebrospinal Fluid Assessment in Treated and Untreated Cohorts **eTable 1. **Associations of Vascular Risk Factors With Aβ Burden Measured With PET and Its Moderation By Vascular Medication Use Additionally Adjusted for *APOE *ε4 Status **eTable 2. **Associations of Vascular Risk Factors With Tau Burden Measured With PET and Its Moderation by Vascular Medication Use Additionally Adjusted for *APOE *ε4 Status **eTable 3. **Associations of Vascular Risk Factors With Aβ1-42 as Measured by Cerebrospinal Fluid Assessment Moderated by Vascular Medication Use **eTable 4. **Associations of Vascular Risk Factors With Aβ1-42 Measured in Cerebrospinal Fluid and Its Moderation by Vascular Medication Use Additionally Adjusted for *APOE *ε4 Status **eTable 5. **Associations of Vascular Risk Factors With Phosphorylated Tau as Measured by Cerebrospinal Fluid Assessment Moderated by Vascular Medication Use **eTable 6. **Associations of Vascular Risk Factors With Phosphorylated Tau Measured in Cerebrospinal Fluid and Its Moderation by Vascular Medication Use Additionally Adjusted for *APOE *ε4 Status
eReferences.
