# Supplementary material for: Association of Vascular Risk Factors With β-Amyloid Peptide and Tau Burdens in Cognitively Unimpaired Individuals and Its Interaction With Vascular Medication Use
Source: JAMA Netw Open. 2020 Feb 7;3(2):e1920780. doi: 10.1001/jamanetworkopen.2019.20780 (PMC12520710; doi:10.1001/jamanetworkopen.2019.20780)
Supplement: Supplement. — eAppendix. Supplementary Methods eFigure 1. Study Flow Chart eFigure 2. Associations of Vascular Risk Factors With Tau Burden as Measured by Positron Emission Tomography in Treated and Untreated Cohorts eFigure 3. Associations of Vascular Risk Factors With Phosphorylated Tau as Measured by Cerebrospinal Fluid Assessment in Treated and Untreated Cohorts eTable 1. Associations of Vascular Risk Factors With Aβ Burden Measured With PET and Its Moderation By Vascular Medication Use Additionally Adjusted for APOE ε4 Status eTable 2. Associations of Vascular Risk Factors With Tau Burden Measured With PET and Its Moderation by Vascular Medication Use Additionally Adjusted for APOE ε4 Status eTable 3. Associations of Vascular Risk Factors With Aβ1-42 as Measured by Cerebrospinal Fluid Assessment Moderated by Vascular Medication Use eTable 4. Associations of Vascular Risk Factors With Aβ1-42 Measured in Cerebrospinal Fluid and Its Moderation by Vascular Medication Use Additionally Adjusted for APOE ε4 Status eTable 5. Associations of Vascular Risk Factors With Phosphorylated Tau as Measured by Cerebrospinal Fluid Assessment Moderated by Vascular Medication Use eTable 6. Associations of Vascular Risk Factors With Phosphorylated Tau Measured in Cerebrospinal Fluid and Its Moderation by Vascular Medication Use Additionally Adjusted for APOE ε4 Status eReferences. [file jamanetwopen-e1920780-s001.pdf]

Supplementary Online Content

Köbe T, Gonneaud J, Pichet Binette A, et al; Presymptomatic Evaluation of Experimental or Novel Treatments for Alzheimer Disease (PREVENT-AD) Research Group. Association of vascular risk factors with β-amyloid peptide and tau burdens in cognitively unimpaired individuals and its interaction with vascular medication use. *JAMA Netw Open*. 2020;3(2):e1920780. doi:10.1001/jamanetworkopen.2019.20780

eAppendix. Supplementary Methods
eFigure 1. Study Flow Chart
eFigure 2. Associations of Vascular Risk Factors With Tau Burden as Measured by Positron Emission Tomography in Treated and Untreated Cohorts
eFigure 3. Associations of Vascular Risk Factors With Phosphorylated Tau as Measured by Cerebrospinal Fluid Assessment in Treated and Untreated Cohorts
eTable 1. Associations of Vascular Risk Factors With Aβ Burden Measured With PET and Its Moderation By Vascular Medication Use Additionally Adjusted for *APOE* ε4 Status
eTable 2. Associations of Vascular Risk Factors With Tau Burden Measured With PET and Its Moderation by Vascular Medication Use Additionally Adjusted for *APOE* ε4 Status
eTable 3. Associations of Vascular Risk Factors With Aβ1-42 as Measured by Cerebrospinal Fluid Assessment Moderated by Vascular Medication Use
eTable 4. Associations of Vascular Risk Factors With Aβ1-42 Measured in Cerebrospinal Fluid and Its Moderation by Vascular Medication Use Additionally Adjusted for *APOE* ε4 Status
eTable 5. Associations of Vascular Risk Factors With Phosphorylated Tau as Measured by Cerebrospinal Fluid Assessment Moderated by Vascular Medication Use
eTable 6. Associations of Vascular Risk Factors With Phosphorylated Tau Measured in Cerebrospinal Fluid and Its Moderation by Vascular Medication Use Additionally Adjusted for *APOE* ε4 Status
eReferences.

This supplementary material has been provided by the authors to give readers additional information about their work.

© 2020 Köbe T et al. *JAMA Network Open.*

eAppendix. Supplementary Methods

Vascular risk factor assessment
All venous blood samples were taken non-fasting at enrollment, because most of each person's lifetime is spent in the postprandial state,[1] lipid profiles change minimally in response to normal food intake, and non-fasting lipid profiles seem to predict increased risk of cardiovascular events better than fasting lipid profiles.[2,3]
Note that new guidelines move towards a consensus on measuring lipid profiles for cardiovascular risk prediction in the non-fasting state.[3] In addition, only triglycerides and LDL-cholesterol (calculated based on triglyceride concentrations, using the Friedewald equation) seem to be influenced by non-fasting state. In the current study, however, triglycerides were not included, and LDL-cholesterol was measured using a direct homogeneous assay.

Blood pressure was additionally measured at most of the annual visits. Participants were informed about abnormal values and the study physician informed their general practitioner if the participant consented. For the current study, to ensure that vascular risk factor measurements were not biased by participants' changes in lifestyle or medication due to an increased awareness after study inclusion, analyses were restricted to vascular risk factor measurements at enrollment (t=0). This research decision is also motivated by increasing evidence suggesting that vascular risk factors earlier on are harmful for later AD risk.[4]

Assessments of Aβ and Tau Burdens
PET scans (amyloid-β, [^{18}F]NAV4694 (NAV) provided by Navidea Biopharmaceuticals (Dublin, Ohio) and *tau*, [^{18}F]AV1451 (Flortaucipir) provided by Eli Lilly & Company (Indianapolis, Indiana)) were acquired ordinarily on two consecutive days. Approximately 6mCi of NAV and 10mCi of Flortaucipir were injected intravenously. Static acquisition frames were obtained for Aβ at 40-70min (6x5min frames) and for *tau* at 80-100min (4x5min frames) post-injection.

CSF samples were obtained with an atraumatic Sprotte needle (24G) via aspiration and stored in polypropylene tubes at −80°C. Collection, storage, and assay techniques were performed as standardized by the European project BIOMARKAPD that was created to harmonize assays used to measure biological markers in neurodegenerative diseases.[5]

Genotyping
APOE genotype was determined using the PyroMark Q96 pyrosequencer (Qiagen, Toronto, ON, Canada) and the following primers: rs429358_amplification_forward 5'-ACGGCTGTCCAAGGAGCT G-3', rs429358_amplification_reverse_biotinylated 5'-CACCTCGCCGCGGTACTG-3', rs429358_sequencing 5'-CGGACATGGAGGACG-3', rs7412_amplification_forward 5'-CTCCGCGATGCCGATGAC-3', rs7412_amplification_reverse_biotinylated 5'-CCCCGGCCTGGTACACTG-3' and rs7412_sequencing 5’-CGATGACCTGCAGAAG-3’; also described previously.[6]

© 2020 Köbe T et al. *JAMA Network Open.*

eFigure 1. Study Flow Chart

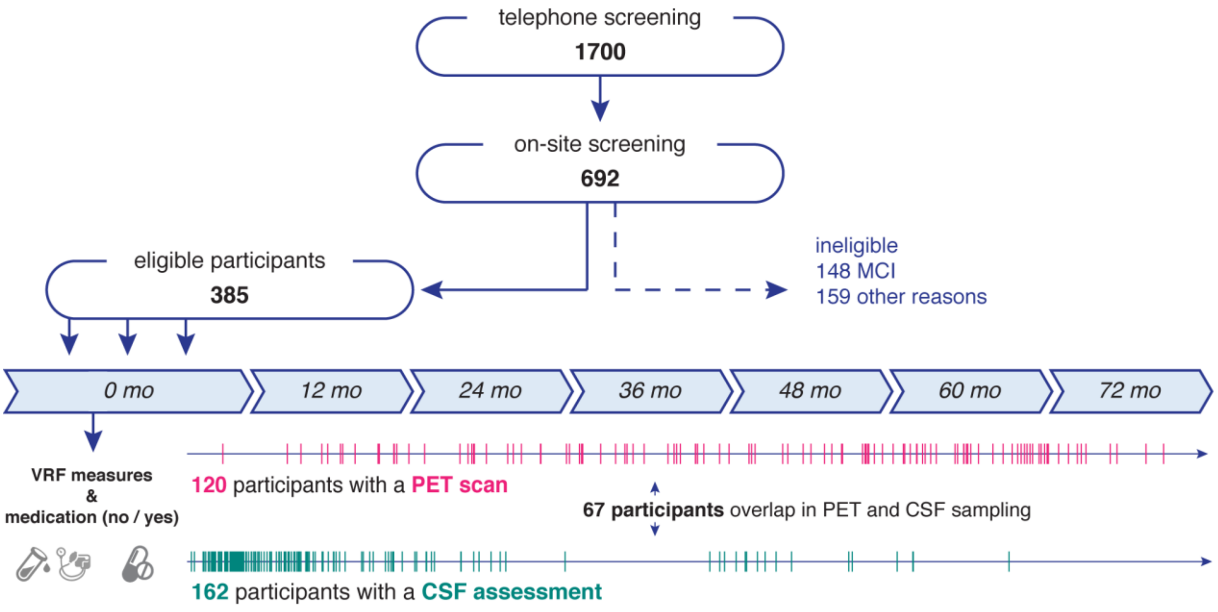

In total, 385 eligible PREVENT-AD participants were followed annually over up to 72 months. For the current cross-sectional analyses, vascular risk factors and vascular medication status were assessed at study enrollment (t=0). During the course of the study, 120 participants obtained Aβ and *tau* scans and 162 participants underwent CSF sampling. The vertical lines display at which time course of the study the PET (magenta) or CSF (cyan) assessments took place (31% participants had both PET and CSF sampling).

© 2020 Köbe T et al. *JAMA Network Open.*

eFigure 2. Associations of Vascular Risk Factors With Tau Burden as Measured by Positron Emission Tomography in Treated and Untreated Cohorts

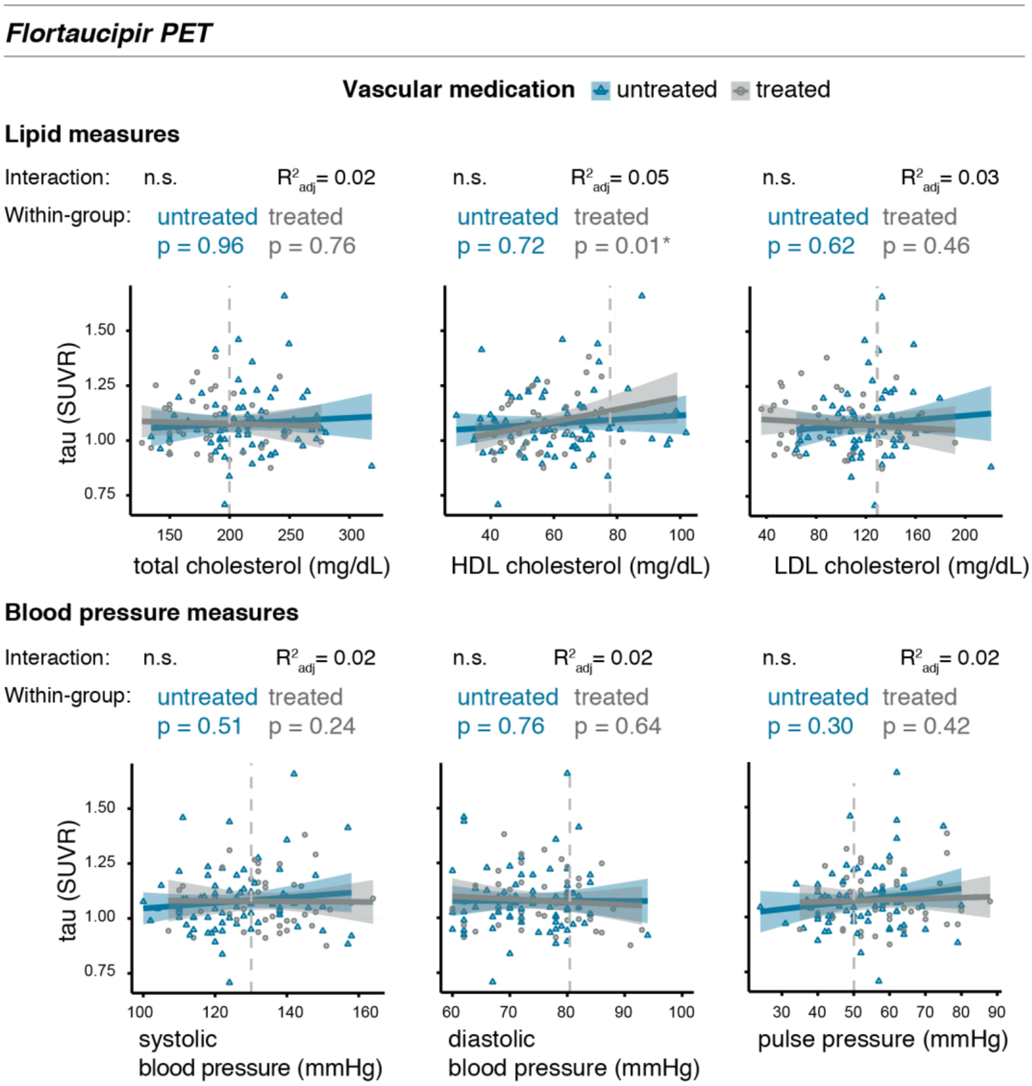

Concentrations of vascular risk factors are plotted against entorhinal tau standard uptake value ratios (SUVR) measured with PET, stratified for individuals with (gray) and without (blue) vascular drug treatment (unadjusted raw data shown). Multiple linear regression analyses were controlled for age, sex and time differences between vascular risk factors and PET assessments. Interaction s between vascular risk factors and vascular medication use and within-group effects are indicated within the graphs ($p<0.05$* and adjusted-R^2). Shaded areas represent 95% CI. Vertical dotted lines indicate thresholds for abnormal vascular risk factor values.

© 2020 Köbe T et al. *JAMA Network Open.*

eFigure 3. Associations of Vascular Risk Factors With Phosphorylated Tau as Measured by Cerebrospinal Fluid Assessment in Treated and Untreated Cohorts

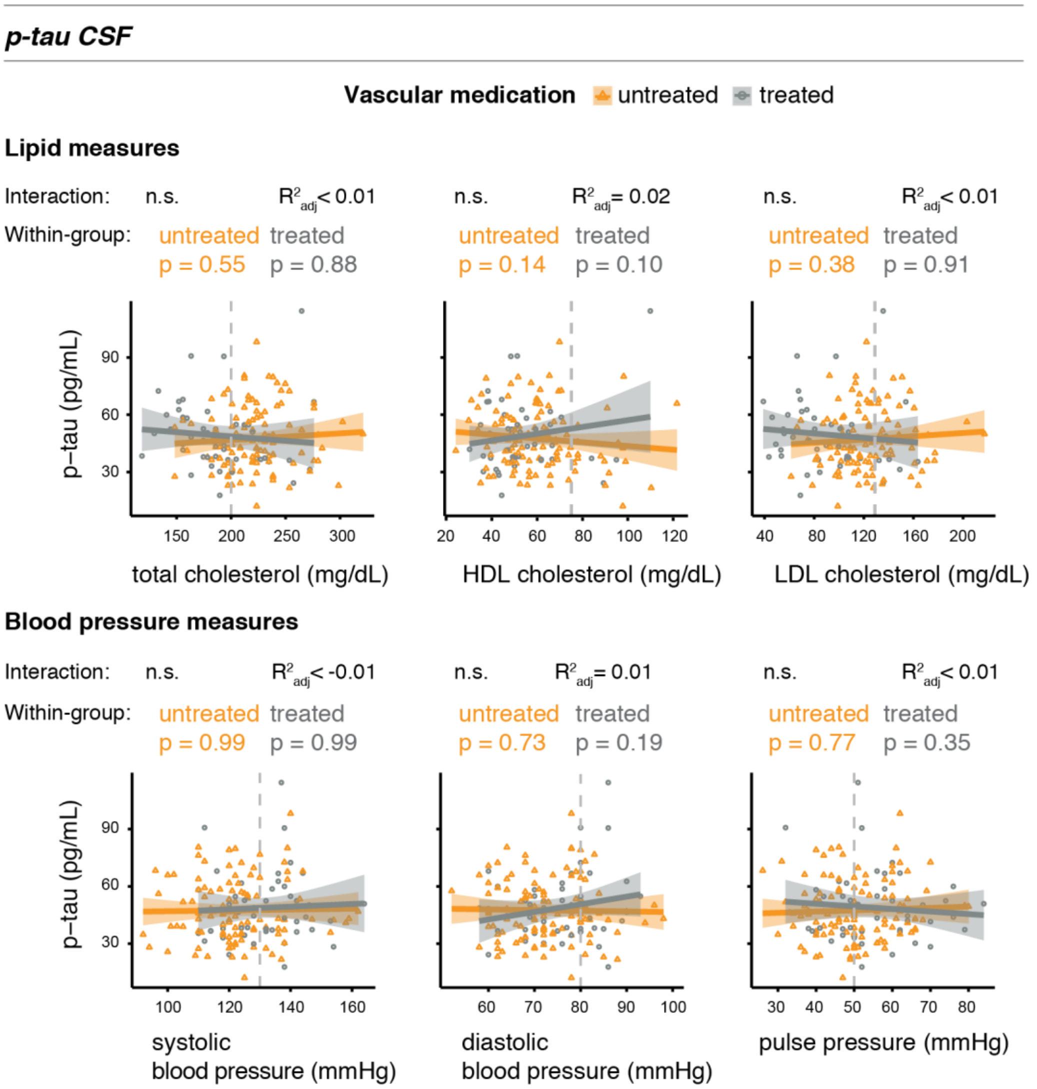

Concentrations of vascular risk factors are plotted against p-tau measured in CSF, stratified for individuals with (gray) and without (orange) vascular drug treatment (unadjusted raw data shown). Multiple linear regression analyses were controlled for age, sex and time differences between vascular risk factors and CSF assessments. Interactions between vascular risk factors and vascular medication use and within-group effects are indicated within the graphs ($p<0.05$* and adjusted-R^2). Shaded areas represent 95% CI. Vertical dotted lines indicate thresholds for abnormal vascular risk factor values.

© 2020 Köbe T et al. *JAMA Network Open.*

eTable 1. Associations of Vascular Risk Factors With Aβ Burden Measured With PET and Its Moderation By Vascular Medication Use Additionally Adjusted for *APOE* ε4 Status

	**Aβ Burden, Unstandardized β (SE), P Value**						
**Variable**	**Cholesterol Level**			**Blood Pressure**		**Pulse Pressure**	**FCRP Score**
	**Total**	**HDL**	**LDL**	**Systolic**	**Diastolic**		
**Total group**							
**Model 1**							
Age	0.017 (0.006), 0.007	0.017 (0.006), 0.006	0.017 (0.006), 0.007	0.014 (0.006), 0.023	0.017 (0.006), 0.007	0.014 (0.006), 0.068	0.016 (0.006), 0.016
Sex	−0.036 (0.065), 0.552	−0.045 (0.067), 0.503	−0.067 (0.068), 0.326	−0.024 (0.063), 0.702	−0.030 (0.063), 0.639	−0.023 (0.063), 0.716	−0.068 (0.069), 0.325
Time difference	−0.002 (0.002), 0.162	−0.002 (0.001), 0.159	−0.002 (0.002), 0.179	−0.002 (0.001), 0.150	−0.002 (0.001), 0.140	−0.002 (0.001), 0.145	−0.002 (0.002), 0.141
Medication	-0.023 (0.063), 0.716	−0.030 (0.059), 0.617	-0.012 (0.068), 0.864	−0.057 (0.060), 0.347	−0.038 (0.059), 0.522	−0.053 (0.059), 0.375	−0.033 (0.060), 0.585
APOE ε4	0.171 (0.058), 0.004	0.174 (0.06), 0.003	0.164 (0.06), 0.008	0.180 (0.056), 0.002	0.176 (0.057), 0.002	0.176 (0.056), 0.002	0.176 (0.058), 0.003
Vascular risk factor	<0.001 (0.001), 0.605	0.001 (0.002), 0.528	0.001 (0.001), 0.552	0.003 (0.002), 0.227	0.001 (0.004), 0.885	0.003 (0.003), 0.205	0.006 (0.012), 0.629
**Model 2[a]**							
Interaction between vascular risk factor and medication use	−0.002 (0.001), 0.041	0.001 (0.002), 0.806	−0.002 (0.001), 0.012	−0.005 (0.002), 0.017	−0.001 (0.004), 0.873	−0.007 (0.002), 0.005	−0.035 (0.011), 0.001
**Untreated cohort**							
Age	0.020 (0.007), 0.006	0.022 (0.007), 0.004	0.020 (0.007), 0.007	0.017 (0.007), 0.020	0.022 (0.007), 0.004	0.016 (0.007), 0.023	0.015 (0.008), 0.054
Sex	0.039 (0.080), 0.629	0.074 (0.086), 0.390	0.033 (0.081), 0.684	0.092 (0.075), 0.277	0.071 (0.078), 0.370	0.110 (0.074), 0.142	0.031 (0.081), 0.704
Time difference	−0.002 (0.002), 0.194	−0.002 (0.002), 0.200	−0.002 (0.002), 0.300	−0.002 (0.002), 0.260	−0.002 (0.002), 0.191	−0.002 (0.002), 0.305	−0.003 (0.002), 0.138
APOE ε4	0.146 (0.073), 0.049	0.177 (0.071), 0.015	0.124 (0.075), 0.105	0.181 (0.067), 0.009	0.175 (0.071), 0.016	0.164 (0.066), 0.015	0.185 (0.070), 0.010
Vascular risk factor	0.001 (0.001), 0.155	<−0.001 (0.002), 0.879	0.003 (0.001), 0.032	0.006 (0.003), 0.014	<−0.001 (0.005), 0.941	0.010 (0.003), 0.002	0.033 (0.015), 0.025
**Treated Cohort**							
Age	0.009 (0.011), 0.424	0.009 (0.011), 0.403	0.010 (0.011), 0.344	0.013 (0.011), 0.269	0.008 (0.011), 0.440	0.013 (0.012), 0.272	0.015 (0.011), 0.186
Sex	−0.163 (0.108), 0.139	−0.202 (0.109), 0.072	−0.242 (0.116), 0.044	−0.183 (0.106), 0.093	−0.184 (0.107), 0.095	−0.179 (0.106), 0.100	−0.198 (0.121), 0.111
Time difference	−0.003 (0.003), 0.273	−0.003 (0.003), 0.361	−0.005 (0.003), 0.135	−0.002 (0.003), 0.426	−0.002 (0.003), 0.379	−0.002 (0.003), 0.454	−0.004 (0.003), 0.183
APOE ε4	0.182 (0.093), 0.057	0.157 (0.097), 0.112	0.159 (0.096), 0.105	0.167 (0.094), 0.082	0.178 (0.094), 0.067	0.172 (0.093), 0.074	0.111 (0.095), 0.246
Vascular risk factor	−0.001 (0.001), 0.348	0.003 (0.004), 0.425	−0.001 (0.001) 0.296	−0.004 (0.004), 0.337	−0.001 (0.006), 0.845	−0.004 (0.004), 0.402	−0.028 (0.019), 0.142

Abbreviation: Aβ, amyloid-β peptide; FCRP, Framingham Coronary Risk Profile; HDL, high-density lipoprotein; LDL, low-density lipoprotein; Unstandardized β, unstandardized regression coefficient.
[a]Included all variables of Model 1 and the interaction between vascular risk factors and medication.

© 2020 Köbe T et al. *JAMA Network Open.*

eTable 2. Associations of Vascular Risk Factors With Tau Burden Measured With PET and Its Moderation by Vascular Medication Use Additionally Adjusted for *APOE* ε4 Status

	**Tau Burden, Unstandardized β (SE), P Value**						
**Variable**	**Cholesterol Level**			**Blood Pressure**		**Pulse Pressure**	**FCRP Score**
	**Total**	**HDL**	**LDL**	**Systolic**	**Diastolic**		
**Total group**							
**Model 1**							
Age	0.008 (0.003), 0.003	0.008 (0.003), 0.003	0.008 (0.003), 0.003	0.008 (0.003), 0.006	0.008 (0.003), 0.003	0.008 (0.003), 0.008	0.010 (0.003), 0.001
Sex	0.024 (0.029), 0.402	0.005 (0.030), 0.873	0.017 (0.030), 0.570	0.020 (0.028), 0.475	0.020 (0.028), 0.478	0.021 (0.028), 0.467	0.025 (0.031), 0.408
Time difference	<−0.001 (0.001), 0.990	<0.001 (0.001), 0.884	<0.001 (0.001), 0.885	<−0.001 (0.001), 0.937	<−0.001 (0.001), 0.947	<−0.001 (0.001), 0.935	<−0.001 (0.001), 0.953
Medication	−0.024 (0.028), 0.392	−0.013 (0.026), 0.626	−0.027 (0.030), 0.374	−0.019 (0.027), 0.487	−0.017 (0.026), 0.517	−0.020 (0.027), 0.462	−0.013 (0.027), 0.632
APOE ε4	0.061 (0.026), 0.019	0.055 (0.025), 0.030	0.059 (0.027), 0.030	0.060 (0.025), 0.019	0.059 (0.025), 0.020	0.060 (0.025), 0.018	0.050 (0.026), 0.054
Vascular risk factor	<−0.001 (<0.001), 0.609	0.001 (0.001), 0.110	<−0.001 (0.001), 0.486	<0.001 (0.001), 0.896	−0.001 (0.002), 0.846	<0.001 (0.001), 0.774	−0.008 (0.005), 0.124
**Model 2[a]**							
Interaction between vascular risk factor and medication use	<−0.001 (<0.001), 0.761	0.001 (0.001), 0.449	<0.001 (<0.001), 0.495	−0.001 (0.001), 0.425	<0.001 (0.002), 0.858	−0.001 (0.001), 0.445	−0.009 (0.005), 0.068
**Untreated cohort**							
Age	0.008 (0.004), 0.035	0.007 (0.004), 0.038	0.008 (0.004), 0.037	0.007 (0.004), 0.060	0.008 (.004), 0.034	0.007 (0.004), 0.066	0.008 (0.004), 0.051
Sex	0.070 (0.039), 0.079	0.060 (0.042), 0.157	0.064 (0.040), 0.116	0.067 (0.038), 0.082	0.064 (0.038), 0.096	0.070 (0.038), 0.070	0.064 (0.041), 0.117
Time difference	<0.001 (0.001), 0.842	<0.001 (0.001), 0.810	<0.001 (0.001), 0.824	<0.001 (0.001), 0.755	<0.001 (0.001), 0.808	<0.001 (0.001), 0.721	<0.001 (0.001), 0.820
APOE ε4	0.062 (0.036), 0.088	0.057 (0.034), 0.099	0.054 (0.038), 0.158	0.061 (0.034), 0.079	0.059 (0.034), 0.089	0.058 (0.034), 0.091	0.053 (0.035), 0.136
Vascular risk factor	<0.001 (<0.001), 0.650	<0.001 (0.001), 0.718	<0.001 (0.001), 0.958	0.001 (0.001), 0.459	<0.001 (0.002), 0.942	0.002 (0.002), 0.332	<0.001 (0.007), 0.980
**Treated Cohort**							
Age	0.010 (0.004), 0.020	0.010 (0.004), 0.013	0.011 (0.004), 0.010	0.012 (0.005), 0.014	0.010 (0.004), 0.023	0.011 (0.005), 0.022	0.014 (0.004), 0.001
Sex	−0.053 (0.041), 0.198	−0.076 (0.038), 0.054	−0.078 (0.043), 0.076	−0.055 (0.040), 0.169	−0.057 (0.040), 0.161	−0.055 (0.040), 0.176	−0.043 (0.042), 0.315
Time difference	−0.001 (0.001), 0.596	−0.001 (0.001), 0.562	−0.001 (0.001), 0.198	<0.001 (0.001), 0.693	<0.001 (0.001), 0.632	<0.001 (0.001), 0.712	−0.001 (0.001), 0.204
APOE ε4	0.061 (0.035), 0.090	0.040 (0.034), 0.243	0.052 (0.036), 0.151	0.055 (0.035), 0.131	0.060 (0.035), 0.097	0.058 (0.035), 0.109	0.025 (0.034), 0.459
Vascular risk factor	<0.001 (0.001), 0.730	0.003 (0.001), 0.026	<0.001 (<0.001), 0.323	−0.002 (0.002), 0.370	−0.001 (0.002), 0.715	−0.001 (0.002), 0.548	−0.018 (0.007), 0.012

Abbreviation: FCRP, Framingham Coronary Risk Profile; HDL, high-density lipoprotein; LDL, low-density lipoprotein; Unstandardized β, unstandardized regression coefficient. [a]Included all variables of Model 1 and the interaction between vascular risk factors and medication.

© 2020 Köbe T et al. *JAMA Network Open.*

eTable 3. Associations of Vascular Risk Factors With Aβ1-42 as Measured by Cerebrospinal Fluid Assessment Moderated by Vascular Medication Use

	Aβ1-42 Burden, Unstandardized β (SE), P Value						
**Variable**	**Cholesterol Level**			**Blood Pressure**		**Pulse Pressure**	**FCRP Score**
	**Total**	**HDL**	**LDL**	**Systolic**	**Diastolic**		
**Total group**							
**Model 1**							
Age	-2.5 (4.2), 0.551	-1.5 (4.3), 0.733	-1.4 (4.3), 0.751	-1.8 (4.4), 0.682	-2.6 (4.4), 0.561	-2 (4.5), 0.658	-0.01 (4.5), 0.999
Sex	143.4 (51.5), 0.006	150.9 (55.0), 0.007	129.6 (51.7), 0.014	89.4 (51.4), 0.084	90. 7 (51.2), 0.078	92.5 (51.3), 0.074	110.2 (54.0), 0.043
Time difference	2.6 (1.9) 0.168	1.8 (2.0), 0.369	2.7 (1.9), 0.158	2.5 (1.9), 0.207	2.5 (1.9), 0.205	2.4 (1.9), 0.223	2.8 (2.0), 0.167
Medication	-116.8 (52.7), 0.028	-71.1 (50.6), 0.163	-134.7 (56.6), 0.019	-48.1 (52.6), 0.361	-51.0 (51.8), 0.327	-54.3 (52.2), 0.300	-66.3 (52.3), 0.207
Vascular risk factor	-2.01 (0.64), 0.002	-3.53 (1.47), 0.018	-2.12 (0.8), 0.096	-1.3 (1.7), 0.419	-2.4 (2.7), 0.367	-0.7 (2.1), 0.751	-5.6 (10.2), 0.581
**Model 2[a]**							
Interaction between vascular risk factor and medication use	0.82 (0.63), 0.195	-3.30 (1.5), 0.034	1.55 (0.75), 0.042	1.7 (1.9), 0.376	-1.1 (2.9), 0.716	3.1 (2.1), 0.147	23.1 (10.9), 0.038
**Untreated cohort**							
Age	-2.1 (4.8), 0.670	-2.3 (5.1), 0.649	-0.82 (4.7), 0.864	-2.1 (5.1), 0.686	-2.8 (5.1), 0.582	-1.6 (5.2), 0.755	1.3 (5.0), 0.802
Sex	180.9 (64.2), 0.006	160.8 (70.3), 0.025	152.1 (60.6), 0.014	113.4 (65.1), 0.085	120.5 (65.3), 0.068	106.7 (65.6), 0.107	151.8 (64.8), 0.021
Time difference	-1.9 (3.2), 0.559	-3.3 (3.4), 0.334	-2.3 (3.1), 0.456	-3.3 (3.3), 0.319	-3.2 (3.3), 0.338	-3.8 (3.3), 0.256	-2.4 (3.2), 0.457
Vascular risk factor	-2.69 (0.81), 0.001	-2.33 (1.7), 0.178	-3.27 (0.9), 0.001	-2.0 (1.9), 0.299	-0.9 (3.4), 0.799	-3.1 (2.6), 0.231	-17.5 (11.4), 0.130
**Treated Cohort**							
Age	-3.6 (8.5), 0.675	-0.35 (7.7), 0.964	-3.2 (9.1), 0.723	-3.1 (8.5), 0.717	-4.8 (8.5), 0.578	-4.9 (8.7), 0.570	-4.1 (8.7), 0.638
Sex	46.8 (84.7), 0.584	145.1 (83.2), 0.089	26.3 (95.1), 0.784	31.9 (80.9), 0.696	5.7 (82.3), 0.945	27.4 (79.5), 0.732	-22.9 (93.3), 0.807
Time difference	5.7 (2.4), 0.020	3.7 (2.3), 0.108	5.8 (2.5), 0.023	5.8 (2.3), 0.020	5.9 (2.3), 0.016	5.6 (2.4), 0.023	4.2 (2.6), 0.124
Vascular risk factor	-0.57 (1.03), 0.582	-7.86 (2.7), 0.007	0.17 (1.3), 0.896	0.23 (3.1), 0.942	-4.9 (4.5), 0.287	2.8 (3.3), 0.398	31.4 (20.9), 0.143

Abbreviation: Aβ, amyloid-β peptide; FCRP, Framingham Coronary Risk Profile; HDL, high-density lipoprotein; LDL, low-density lipoprotein; Unstandardized β, unstandardized regression coefficient.
[a]Included all variables of Model 1 and the interaction between vascular risk factors and medication.

© 2020 Köbe T et al. *JAMA Network Open.*

eTable 4. Associations of Vascular Risk Factors With Aβ1-42 Measured in Cerebrospinal Fluid and Its Moderation by Vascular Medication Use Additionally Adjusted for *APOE* ε4 Status

	Aβ1-42 Burden, Unstandardized β (SE), P Value						
**Variable**	**Cholesterol Level**			**Blood Pressure**		**Pulse Pressure**	**FCRP Score**
	**Total**	**HDL**	**LDL**	**Systolic**	**Diastolic**		
**Total group**							
**Model 1**							
Age	-6.2 (4.1), 0.135	-5.6 (4.2), 0.182	-5.1 (4.2), 0.221	-6.1 (4.3), 0.157	-6.5 (4.4), 0.125	-6.6 (4.4), 0.132	-4.5 (4.3), 0.294
Sex	108.1 (49.8), 0.032	115.5 (52.5), 0.030	95.8 (49.5), 0.055	66.2 (48.7), 0.176	64.9 (48.5), 0.183	68.4 (48.6), 0.162	78.0 (50.9), 0.128
Time difference	2.2 (1.8) 0.228	1.4 (1.8), 0.434	2.2 (1.8), 0.220	1.8 (1.8), 0.318	1.9 (1.8), 0.301	1.8 (1.8), 0.333	2.2 (1.9), 0.241
Medication	-80.0 (51.0), 0.119	-44.8 (48.1), 0.354	-86.5 (54.6), 0.116	-27.7 (49.8), 0.579	-25.4 (49.1), 0.605	-32.6 (49.4), 0.510	-32.5 (49.4), 0.512
APOE ε4	-178.9 (45.5), <0.001	-191.3 (45.1), <0.001	-190.7 (46.2), <0.001	-195.7 (46.1), <0.001	-196.6 (45.6), <0.001	-199.3 (46.1), <0.001	-208.0 (46.3), <0.001
Vascular risk factor	-1.50 (0.62), 0.017	-2.9 (1.40), 0.043	-1.6 (0.7), 0.030	-0.5 (1.6), 0.763	-2.2 (2.5), 0.395	-0.5 (2.0), 0.783	-3.6 (9.5), 0.706
**Model 2**[a]							
Interaction between vascular risk factor and medication use	0.45 (0.61), 0.466	-3.2 (1.5), 0.068	1.1 (0.7), 0.123	1.0 (1.8), 0.592	-1.4 (2.7), 0.599	2.1 (2.0), 0.298	15.7 (10.5), 0.136
**Untreated cohort**							
Age	-4.9 (4.9), 0.321	-5.8 (5.0), 0.250	-3.4 (4.7), 0.476	-5.5 (5.1), 0.282	-6.0 (5.0), 0.236	-5.3 (5.2), 0.306	-2.5 (5.0), 0.623
Sex	153.3 (63.7), 0.018	130.7 (68.1), 0.058	133.7 (59.3), 0.027	97.2 (63.0), 0.126	100.4 (63.0), 0.114	94.9 (63.5), 0.138	128.8 (62.4), 0.042
Time difference	-0.4 (3.2), 0.898	-1.0 (3.3), 0.761	-0.7 (3.0), 0.810	-1.6 (3.2), 0.627	-1.4 (3.3), 0.666	-1.8 (3.2), 0.583	-0.4 (3.2), 0.892
APOE ε4	-139.6 (59.2), 0.020	-176.7 (58.6), 0.003	-142.3 (57.8), 0.016	-169.0 (59.2), 0.005	-174.7 (58.2), 0.003	-167.1 (60.0), 0.006	-178.5 (58.1), 0.003
Vascular risk factor	-2.1 (0.83), 0.014	-1.6 (1.7), 0.330	-2.6 (0.9), 0.005	-1.0 (1.9), 0.601	-0.808 (3.3), 0.807	-1.3 (2.6), 0.605	-13.3 (11.0), 0.231
**Treated Cohort**							
Age	-8.2 (8.2), 0.325	-4.7 (7.4), 0.530	-8.0 (8.7), 0.361	-7.6 (8.2), 0.359	-9.5 (8.2), 0.251	-9.8 (8.3), 0.249	-8.6 (8.4), 0.310
Sex	23.0 (80.2), 0.775	116.4 (78.6), 0.147	12.9 (85.9), 0.887	6.8 (76.9), 0.930	-22.545 (77.8), 0.774	1.1 (75.2), 0.988	-53.0 (88.2), 0.552
Time difference	3.7 (2.4), 0.126	1.9 (2.2), 0.398	3.7 (2.5), 0.143	3.8 (2.4), 0.122	3.8 (2.3), 0.107	3.5 (2.4), 0.146	2.3 (2.6), 0.374
APOE ε4	-201.7 (81.4), 0.018	-189.7 (74.0), 0.014	-222.6 (87.9), 0.016	-200.4 (81.8), 0.019	-204.6 (80.2), 0.015	-203.9 (80.8), 0.016	-212.3 (85.9), 0.018
Vascular risk factor	-0.63 (1.0), 0.516	-7.5 (5.6), 0.096	0.17 (1.2), 0.888	0.31 (2.9), 0.915	-5.4 (4.3), 0.215	3.2 (3.1), 0.314	27.1 (19.7), 0.177

Abbreviation: Aβ, amyloid-β peptide; FCRP, Framingham Coronary Risk Profile; HDL, high-density lipoprotein; LDL, low-density lipoprotein; Unstandardized β, unstandardized regression coefficient.
[a]Included all variables of Model 1 and the interaction between vascular risk factors and medication.

© 2020 Köbe T et al. *JAMA Network Open.*

eTable 5. Associations of Vascular Risk Factors With Phosphorylated Tau as Measured by Cerebrospinal Fluid Assessment Moderated by Vascular Medication Use

	Phosphorylated Tau Burden, Unstandardized β (SE), P Value						
**Variable**	**Cholesterol Level**			**Blood Pressure**		**Pulse Pressure**	**FCRP Score**
	**Total**	**HDL**	**LDL**	**Systolic**	**Diastolic**		
**Total group**							
**Model 1**							
Age	0.37 (0.27), 0.161	0.38 (0.27), 0.157	0.37 (0.27), 0.175	0.36 (0.27), 0.176	0.38 (0.27), 0.158	0.39 (0.27), 0.153	0.38 (0.27), 0.169
Sex	-1.8 (3.1), 0.577	-1.2 (3.3), 0.724	-2.8 (3.1), 0.365	-1.2 (3), 0.693	-0.97 (3.0), 0.745	-1.3 (3.0), 0.655	-2.2 (3.2), 0.481
Time difference	0.16 (0.1), 0.120	0.16 (0.1), 0.133	0.17 (0.1), 0.104	0.16 (0.1), 0.122	0.15 (0.1), 0.132	0.16 (0.1), 0.110	0.17 (0.1), 0.099
Medication	0.13 (3.2), 0.968	-0.28 (3.0), 0.927	1.2 (3.4), 0.727	-0.45 (3.0), 0.883	-0.67 (3), 0.824	-0.22 (3.0), 0.942	0.518 (3.1), 0.866
Vascular risk factor	0.01 (0.04), 0.732	-0.02 (0.9), 0.851	0.021 (0.05), 0.643	-0.002 (0.1), 0.983	0.1 (0.15), 0.514	-0.07 (0.12), 0.587	-0.29 (0.6), 0.632
**Model 2[a]**							
Interaction between vascular risk factor and medication use	-0.03 (0.04), 0.437	0.16 (0.9), 0.082	-0.04 (0.05), 0.383	0.01 (0.11), 0.959	0.26 (0.17), 0.117	-0.13 (0.12), 0.295	-0.79 (0.67), 0.240
**Untreated cohort**							
Age	0.42 (0.3), 0.161	0.47 (0.29), 0.110	0.4 (0.3), 0.178	0.43 (0.29), 0.146	0.42 (0.29), 0.152	0.42 (0.307), 0.164	0.39 (0.3), 0.200
Sex	0.42 (3.8), 0.912	3.5 (3.9), 0.381	-0.04 (3.7), 0.991	0.99 (3.7), 0.788	0.97 (3.6), 0.791	1.2 (3.7), 0.746	0.23 (3.8), 0.950
Time difference	0.2 (0.15), 0.180	0.18 (0.15), 0.217	0.22 (0.15), 0.140	0.19 (0.15), 0.199	0.2 (0.15), 0.182	0.19 (0.15), 0.196	0.21 (0.15), 0.153
Vascular risk factor	0.03 (0.5), 0.553	-0.15 (0.1), 0.136	0.05 (0.06), 0.376	0 (0.11), 0.998	-0.06 (0.18), 0.727	0.04 (0.15), 0.771	0.03 (0.66), 0.962
**Treated Cohort**							
Age	0.22 (0.61), 0.721	0.13 (0.58), 0.826	0.31 (0.63), 0.624	0.14 (0.6), 0.813	0.26 (0.59), 0.664	0.26 (0.6), 0.668	0.36 (0.62), 0.569
Sex	-5.3 (5.8), 0.373	-10.6 (5.9), 0.077	-7.5 (6.15), 0.228	-4.7 (5.4), 0.389	-2.9 (5.4), 0.590	-4.5 (5.3), 0.398	-5.9 (6.3), 0.349
Time difference	0.14 (0.16), 0.379	0.2 (0.15), 0.202	0.13 (0.16), 0.403	0.15 (0.16), 0.341	0.16 (0.15), 0.295	0.18 (0.16), 0.255	0.18 (0.17), 0.298
Vascular risk factor	-0.01 (0.7), 0.876	0.36 (0.2), 0.065	-0.01 (0.09), 0.909	0.002 (0.22), 0.992	0.41 (0.31), 0.189	-0.21 (0.23), 0.350	-1 (1.4), 0.470

Abbreviation: FCRP, Framingham Coronary Risk Profile; HDL, high-density lipoprotein; LDL, low-density lipoprotein; Unstandardized β, unstandardized regression coefficient. [a]Included all variables of Model 1 and the interaction between vascular risk factors and medication.

© 2020 Köbe T et al. *JAMA Network Open.*

eTable 6. Associations of Vascular Risk Factors With Phosphorylated Tau Measured in Cerebrospinal Fluid and Its Moderation by Vascular Medication Use Additionally Adjusted for *APOE* ε4 Status

	Phosphorylated Tau Burden, Unstandardized β (SE), P Value						
**Variable**	**Cholesterol Level**			**Blood Pressure**		**Pulse Pressure**	**FCRP Score**
	**Total**	**HDL**	**LDL**	**Systolic**	**Diastolic**		
**Total group**							
**Model 1**							
Age	0.4 (0.3), 0.108	0.5 (0.3), 0.099	0.4 (0.3), 0.152	0.4 (0.3), 0.120	0.44 (0.3), 0.112	0.5 (0.3), 0.099	0.4 (0.3), 0.141
Sex	-1.2 (3.2), 0.709	-0.5 (3.3), 0.870	-2.6 (3.2), 0.415	-0.8 (3.0), 0.782	-0.6 (3.0), 0.838	-1.0 (3.0), 0.747	-2.0 (3.2), 0.531
Time difference	0.16 (0.1), 0.127	0.2 (0.1), 0.142	0.2 (0.1), 0.109	0.2 (0.1), 0.120	0.2 (0.1), 0.133	0.2 (0.1), 0.108	0.2 (0.1), 0.102
Medication	-0.5 (3.2), 0.890	-0.7 (3.0), 0.818	0.8 (3.5), 0.809	-0.8 (3.1), 0.804	-1.0 (3.0), 0.736	-0.6 (3.0), 0.851	0.3 (3.1), 0.931
APOE ε4	3.0 (2.9), 0.302	3.2 (2.8), 0.264	1.5 (2.9), 0.608	2.9 (2.8), 0.309	2.7 (2.8), 0.329	3.0 (2.8), 0.278	1.7 (2.9), 0.559
Vascular risk factor	0.01 (0.04), 0.893	-0.03 (0.09), 0.728	0.02 (0.05), 0.712	-0.02 (0.1), 0.878	0.1 (0.2), 0.547	-0.08 (0.12), 0.500	-0.29 (0.6), 0.632
**Model 2[a]**							
Interaction between vascular risk factor and medication use	-0.03 (0.04), 0.536	0.16 (0.9), 0.088	-0.04 (0.05), 0.424	0.02 (0.11), 0.867	0.27 (0.17), 0.106	-0.12 (0.13), 0.360	-0.74 (0.68), 0.279
**Untreated cohort**							
Age	0.4 (0.3), 0.203	0.5 (0.3), 0.116	0.3 (0.3), 0.267	0.4 (0.3), 0.164	0.4 (0.3), 0.167	0.4 (0.3), 0.192	0.3 (0.3), 0.271
Sex	0.2 (3.9), 0.956	3.6 (4.0), 0.379	-0.43 (3.7), 0.908	1.0 (3.7), 0.792	0.9 (3.7), 0.795	1.2 (3.7), 0.753	0.01 (3.8), 0.999
Time difference	0.2 (0.2), 0.173	0.18 (0.2), 0.241	0.2 (0.2), 0.107	0.2 (0.1), 0.205	0.2 (0.2), 0.190	0.2 (0.1), 0.199	0.2 (0.2), 0.135
APOE ε4	-1.0 (3.5), 0.784	0.4 (3.4), 0.899	-2.8 (3.6), 0.434	-1.0 (3.4), 0.967	-0.1 (3.3), 0.983	-0.4 (3.4), 0.914	-1.7 (3.5), 0.615
Vascular risk factor	0.03 (0.1), 0.518	-0.2 (0.1), 0.137	0.1 (0.1), 0.285	0.001 (0.1), 0.992	-0.1 (0.2), 0.729	0.05 (0.2), 0.760	0.07 (0.7), 0.919
**Treated Cohort**							
Age	0.46 (0.6), 0.454	0.3 (0.6), 0.564	0.5 (0.6), 0.436	0.3 (0.6), 0.585	0.5 (0.6), 0.433	0.5 (0.6), 0.456	0.5 (0.6), 0.414
Sex	-4.1 (5.7), 0.480	-8.8 (5.9), 0.141	-6.2 (6.1), 0.322	-3.2 (5.4), 0.551	-1.3 (5.4), 0.811	-3.1 (5.3), 0.563	-5.1 (6.2), 0.414
Time difference	0.2 (0.2), 0.200	0.2 (0.2), 0.116	0.2 (0.2), 0.253	0.2 (0.2), 0.193	0.2 (0.2), 0.149	0.2 (0.2), 0.134	0.2 (0.2), 0.215
APOE ε4	1.0 (5.6), 0.082	8.6 (5.5), 0.125	8.3 (5.9), 0.167	9.0 (5.6), 0.113	9.5 (5.4), 0.089	9.0 (5.5), 0.107	7.8 (6.0), 0.200
Vascular risk factor	-0.01 (0.7), 0.941	0.3 (0.2), 0.105	-0.01 (0.1), 0.955	0.01 (0.2), 0.952	0.4 (0.3), 0.149	-0.2 (0.2), 0.331	-0.7 (1.4), 0.631

© 2020 Köbe T et al. *JAMA Network Open.*

Abbreviation: FCRP, Framingham Coronary Risk Profile; HDL, high-density lipoprotein; LDL, low-density lipoprotein; Unstandardized β, unstandardized regression coefficient. [a]Included all variables of Model 1 and the interaction between vascular risk factors and medication.

© 2020 Köbe T et al. *JAMA Network Open.*

eReferences

1. Rifai N, Young IS, Nordestgaard BG, et al. Nonfasting Sample for the Determination of Routine Lipid Profile: Is It an Idea Whose Time Has Come? *Clin Chem.* 2016;62(3):428-435.
2. Fatima S, Ijaz A, Sharif TB, Khan DA, Siddique A. Accuracy of Non-Fasting Lipid Profile for the Assessment of Lipoprotein Coronary Risk. *J Coll Physicians Surg Pak.* 2016;26(12):954-957.
3. Langsted A, Nordestgaard BG. Nonfasting versus fasting lipid profile for cardiovascular risk prediction. *Pathology.* 2019;51(2):131-141.
4. Gottesman RF, Schneider AL, Zhou Y, et al. Association Between Midlife Vascular Risk Factors and Estimated Brain Amyloid Deposition. *JAMA.* 2017;317(14):1443-1450.
5. Palmqvist S, Zetterberg H, Blennow K, et al. Accuracy of brain amyloid detection in clinical practice using cerebrospinal fluid beta-amyloid 42: a cross-validation study against amyloid positron emission tomography. *JAMA Neurol.* 2014;71(10):1282-1289.
6. Meyer PF, Savard M, Poirier J, et al. Bi-directional Association of Cerebrospinal Fluid Immune Markers with Stage of Alzheimer's Disease Pathogenesis. *J Alzheimers Dis.* 2018;63(2):577-590.

© 2020 Köbe T et al. *JAMA Network Open.*
